# Mutation analysis of *P73* and *TP53* in Merkel cell carcinoma

**DOI:** 10.1054/bjoc.1999.1006

**Published:** 2000-02

**Authors:** M Van Gele, M Kaghad, J H Leonard, N Van Roy, J M Naeyaert, M L Geerts, S Van Belle, V Cocquyt, J Bridge, R Sciot, C De Wolf-Peeters, A De Paepe, D Caput, F Speleman

**Affiliations:** Departments of Medical Genetics, Dermatology and Medical Oncology, University Hospital, Ghent, De Pintelaan 185, Ghent, B-9000, Belgium; Department of Pathology, University Hospital, Minderbroederstraat 12, Leuven, B-3000, Belgium; Queensland Radium Institute Laboratory, Queensland Institute of Medical Research, Herston Road, Brisbane, Queensland, 4029, Australia; Sanofi Recherche, Innopole BP 137, Labege cedex, 31676, France; Department of Human Genetics, University of Nebraska Medical Center, Omaha, NE, 68198–5440, USA

**Keywords:** Merkel cell carcinoma, 1p36;*p73*;*TP53*, mutation

## Abstract

The *p73* gene has been mapped to 1p36.33, a region which is frequently deleted in a wide variety of neoplasms including tumours of neuroectodermal origin. The p73 protein shows structural and functional homology to p53. For these reasons, *p73* was considered as a positional and functional candidate tumour suppressor gene. Thus far, mutation analysis has provided no evidence for involvement of *p73* in oligodendrogliomas, lung carcinoma, oesophageal carcinoma, prostatic carcinoma and hepatocellular carcinoma. In neuroblastoma, two mutations have been observed in a series of 140 tumours. In view of the occurrence of 1p deletions in Merkel cell carcinoma (MCC) and the location of *p73* we decided to search for mutations in the *p73* gene in five MCC cell lines and ten MCC tumours to test potential tumour suppressor function for this gene in MCC. In view of the possible complementary functions of *p73* and *TP53* we also examined the status of the *TP53* gene. Sequence analysis of the entire coding region of the *p73* gene revealed previously reported polymorphisms in four MCCs. In one MCC tumour, a mis-sense mutation located in the NH_2_-terminal transactivation region of the *p73* gene was found. These results show that *p73*, analogous to neuroblastoma, is infrequently mutated in MCC. This is also the first report in which the role of *TP53* in MCC has been investigated by sequencing the entire coding region of *TP53*. *TP53* mis-sense mutations and one non-sense mutation were detected in three of 15 examined MCCs, suggesting that *TP53* mutations may play a role in the pathogenesis or progression of a subset of MCCs. Moreover, typical UVB induced C to T mutations were found in one MCC cell line thus providing further evidence for sun-exposure in the aetiology of this rare skin cancer. © 2000 Cancer Research Campaign


British Journal of Cancer (2000) **82**(4), 823–826
© 2000 Cancer Research Campaign
DOI: 10.1054/ bjoc.1999.1006, available online at http://www.idealibrary.com on IDEAL®

### Mutation analysis of *P73* and *TP53* in Merkel cell carcinoma

**M Van Gele[1], M Kaghad[6], JH Leonard[5], N Van Roy[1], JM Naeyaert[2], ML Geerts[2], S Van Belle[3], V Cocquyt[3], J Bridge[7], R Sciot[4], C De Wolf-Peeters[4], A De Paepe[1], D Caput[6] and F Speleman[1]**

Departments of [1]Medical Genetics, [2]Dermatology and [3]Medical Oncology, University Hospital, Ghent, De Pintelaan 185, B-9000 Ghent, Belgium; [4]Department of Pathology, University Hospital, Minderbroederstraat 12, B-3000 Leuven, Belgium; [5]Queensland Radium Institute Laboratory, Queensland Institute of Medical Research, Herston Road, Brisbane, 4029, Queensland, Australia; [6]Sanofi Recherche, Innopole BP 137, 31676 Labege cedex, France; [7]Department of Human Genetics, University of Nebraska Medical Center, Omaha, NE 68198–5440, USA

**Summary** The *p73* gene has been mapped to 1p36.33, a region which is frequently deleted in a wide variety of neoplasms including tumours of neuroectodermal origin. The p73 protein shows structural and functional homology to p53. For these reasons, *p73* was considered as a positional and functional candidate tumour suppressor gene. Thus far, mutation analysis has provided no evidence for involvement of *p73* in oligodendrogliomas, lung carcinoma, oesophageal carcinoma, prostatic carcinoma and hepatocellular carcinoma. In neuroblastoma, two mutations have been observed in a series of 140 tumours. In view of the occurrence of 1p deletions in Merkel cell carcinoma (MCC) and the location of *p73* we decided to search for mutations in the *p73* gene in five MCC cell lines and ten MCC tumours to test potential tumour suppressor function for this gene in MCC. In view of the possible complementary functions of *p73* and *TP53* we also examined the status of the *TP53* gene. Sequence analysis of the entire coding region of the *p73* gene revealed previously reported polymorphisms in four MCCs. In one MCC tumour, a mis-sense mutation located in the $NH_2$-terminal transactivation region of the *p73* gene was found. These results show that *p73*, analogous to neuroblastoma, is infrequently mutated in MCC. This is also the first report in which the role of *TP53* in MCC has been investigated by sequencing the entire coding region of *TP53*. *TP53* mis-sense mutations and one non-sense mutation were detected in three of 15 examined MCCs, suggesting that *TP53* mutations may play a role in the pathogenesis or progression of a subset of MCCs. Moreover, typical UVB induced C to T mutations were found in one MCC cell line thus providing further evidence for sun-exposure in the aetiology of this rare skin cancer. © 2000 Cancer Research Campaign

**Keywords:** Merkel cell carcinoma; 1p36; *p73*; *TP53*; mutation

Merkel cell carcinoma (MCC) is a rare aggressive neuroendocrine skin tumour, mostly affecting elderly individuals. The majority of primary MCCs occur on sun-exposed areas of the skin, implicating UV exposure in its aetiology. The tumour is thought to originate from Merkel cells, which have properties of both epithelial and neuroendocrine cells. They are located in the basal epidermis and are likely to play a role in signal transduction as receptors for mechanical stimuli in all animals (Ratner et al, 1993).

Until now, little was known about the genetic basis of MCC. Only 13 cytogenetic studies have been reported in the literature (Mitelman et al, 1998). No consistent translocations have been observed but structural abnormalities involving the short arm of chromosome 1 have been noted in 40% of all examined cases. Chromosome region 1p36, which is frequently deleted in a variety of neoplasms including neuroendocrine tumours such as neuroblastoma, is often affected in MCC tumours with 1p rearrangements (Van Gele et al, 1998). Several candidate tumour suppressor genes have been mapped to 1p36 but as yet no direct involvement of these genes in tumours with 1p36 loss could be found (Schwab et al, 1996).

The *p73* gene, located on 1p36.33, has also been considered as a candidate tumour suppressor gene. *p73* shows significant sequence similarity to the transactivation, DNA-binding and oligomerization domain of *TP53* (Kaghad et al, 1997). Jost et al (1997) showed that overexpression of *p73* can activate the transcription of *TP53*-responsive genes like *p21*$^{wafl/cipl}$ and inhibit cell growth in the same way as *TP53* by inducing apoptosis. Mutation analyses for the *p73* gene have been performed in lung carcinoma, oligodendrogliomas, prostatic carcinoma, neuroblastomas, colorectal carcinoma, oesophageal carcinoma and hepatocellular carcinoma (Kovalev et al, 1998; Mai et al, 1998*a*, 1998*b*; Nimura et al, 1998; Nomoto et al, 1998; Sunahara et al, 1998; Takahashi et al, 1998; Ichimiya et al, 1999; Mihara et al, 1999). Except for two mutations in primary neuroblastomas resulting in amino acid substitutions in the C-terminal region of p73 (Ichimiya et al, 1999), no other mutations have been reported.

We decided to perform mutation analysis of the *p73* gene in order to investigate the possible involvement of *p73* in MCC oncogenesis. Five MCC cell lines and ten MCC tumours with known 1p status were examined for mutations in the *p73* gene by direct sequence analysis. In view of the possible complementary functions of *p73* and *TP53*, the status of *TP53* was also investigated.

*Received 9 June 1999*
*Revised 8 September 1999*
*Accepted 9 September 1999*

*Correspondence to:* F Speleman

### MATERIALS AND METHODS

### Samples

Tumour samples were collected at the University Hospitals of Ghent and Leuven, Belgium and the University of Nebraska

Medical Center, Ohama, NE, USA. One part of the sample was snap-frozen in liquid nitrogen and another part was stored at –80°C until analysis. MCC tumour cell line UISO was kindly provided by Dr SG Ronan and Prof Dr TK Das Gupta (Illinois, USA). Cell line MKL-1 was given by Prof Dr ST Rosen (Illinois, USA). Cell lines MCC13, MCC14/2 and MCC26 were a gift from Dr JH Leonard (Queensland, Australia). All cell lines were grown in RPMI-1640 medium (Gibco-BRL, 52400-025). DNA of the tumour samples and cell lines was prepared by standard proteinase K digestion, phenol–chloroform extraction, and ethanol precipitation protocols. Constitutional DNA of the patients was isolated by using the Qiagen blood and cell culture Midi Kit (Westburg, 13343). RNA extraction from the tumour samples and cell lines was done with RNeasy Midi Kits (Westburg, 75144).

### Fluorescence in situ hybridization

In order to determine the copy number of chromosome 1 and presence or absence of 1p deletions, fluorescence in situ hybridization (FISH) was performed on metaphases or interphase nuclei according to Van Roy et al (1994). DNA probes used were pUC1.77 (*D1Z1*) for the heterochromatic region of chromosome 1 (1q12) and p1–79 (*D1Z2*) for the subtelomeric region of chromosome 1 (1p36.33).

### Reverse transcriptase polymerase chain reaction and sequencing analysis cDNA synthesis was performed using 5 μg of total RNA incubated in a 20 μl volume reaction containing 50 mM Tris–HCl (pH 8.3), 10 mM dithiothreitol (DTT), 10 mM potassium chloride (KCl), 0.5 mM dNTP, 20 U RNAsin (Promega), 150 U Super-script II Reverse Transcriptase (Gibco-BRL) and 50 ng $\mu l^{-1}$ oligo-d(T) primer for 1 h at 37°C. The primer pairs sense: 5′-AGGGGACGCAGCGAAACC; antisense: 5′-GGCAGCTTG-GGTCTCTGG; and sense: 5′-GGTGACACGCTTCCCTG; antisense: 5′-GGTGGGAGGCTGTCAGTG were used to amplify the entire coding region of *p73* and *TP53* respectively by reverse transcription polymerase chain reaction (RT-PCR). Long range PCR was performed using the Expand™ Long Template PCR system (Boehringer Mannheim). PCR reactions were performed in a 50 μl reaction volume containing 2 μl of reverse transcriptase products, 500 μM dNTP, 300 nM primers, 10% dimethylsulphoxide, 5 μl of buffer 1 and 3.5 units of *Taq* and Pwo enzyme mix. The PCR amplification consisted of 35 cycles of 95°C for 0.5 min, 58°C for 1 min, 68°C for 2.5 min after starting with a denaturing step at 95°C for 1 min and ending at 68°C for 10 min. The PCR products were purified by spin dialysis sequentially on S400 and P10 resins. Subsequently, the PCR products were directly completely sequenced on an ABI PRISM™ 377 sequencer (Perkin-Elmer) using specific nested sequencing primers and the Big Dye Terminator Sequencing Kit. Cases 6, 7, 8, 9 and 10 (see Table 1) were sequenced on an ALF™ Express automated DNA sequencer (Pharmacia) using a Thermo Sequenase fluorescent labelled primer cycle sequencing kit with 7-deaza-dGTP (Amersham, RPN 2438). To confirm the *p73* nucleotide change found in case 4 the following primer pair sense: 5′-CCAGTTCAATCTGCTGA-GCAG-3′ and antisense: 5′-CCACTTTGAGGTCACTTTCC-3′, located in exon 4, were used to amplify genomic tumour DNA and constitutional DNA of the patient. PCR conditions were the same as those described above. The PCR products were sequenced using the same primers as those to amplify genomic DNA.

**Table 1** Summary of mutation analysis of *p73* and *TP53* in Merkel cell carcinoma

| Cell lines | 1pΔ[a] | Expressed allele(s)[b] | p73 Transcript sequence | AA change | TP53 Transcript sequence | AA change |
|---|---|---|---|---|---|---|
| UISO | No[c] | G/C | wt | – | wt | – |
| MKL-1 | No | G/C | wt | – | wt | – |
| MCC13 | No | G/C;A/T | wt | – | mut 857C>T;858C>T | S241F |
| | | | | | mut 967C>T | P278S |
| MCC14/2 | No | G/C | wt | – | mut950T>A | V272E |
| MCC26 | No | G/C | wt | – | N/A[d] | |
| **MCC samples** | | | | | | |
| Case 1 | No | G/C;A/T | 1118C>T;1157T>C[e] | No | wt | – |
| | | | 1781G>A;1940G>A | No | | |
| Case 2 | Yes | A/T | 1118C>T;1157T>C | No | wt | – |
| | | | 1781G>A;1940G>A | No | | |
| Case 3 | No | G/C;A/T | 1118C>T;1157T>C | No | wt | – |
| | | | 1781G>A;1940G>A | | | |
| Case 4 | No | G/C;A/T | mut 439C>T | S110L | mut550A>T | K139X |
| Case 5 | Yes | G/C;A/T | 1118C>T;1157T>C | No | wt | – |
| | | | 1781G>A;1940G>A | | | |
| Case 6 | NT | G/C | wt | – | wt | – |
| Case 7 | No | G/C;A/T | wt | – | wt | – |
| Case 8 | No | G/C;A/T | wt | – | wt | – |
| Case 9 | No | G/C;A/T | wt | – | wt | – |
| Case 10 | No | G/C;A/T | wt | – | wt | – |

[a] 1pΔ, deletion in the short arm of chromosome 1 determined by fluorescence in situ hybridization with the telomeric D1Z2 probe performed on chromosome metaphases or interphase nuclei. NT: not tested. [b] Refers to the polymorphism of G/C and A/T alleles in exon 2, as determined by RT-PCR on mRNA and subsequent sequencing of products. [c] UISO carries an insertion on 1p36.2 of unknown origin (Van Gele et al, 1998). [d] NA, not available due to lack of *TP53* mRNA expression, as determined by RT-PCR. [e]Refers to the tightly linked polymorphisms located in exon 9 and exon 14, as determined by RT-PCR on mRNA and subsequent sequencing of products.

*© 2000 Cancer Research Campaign*

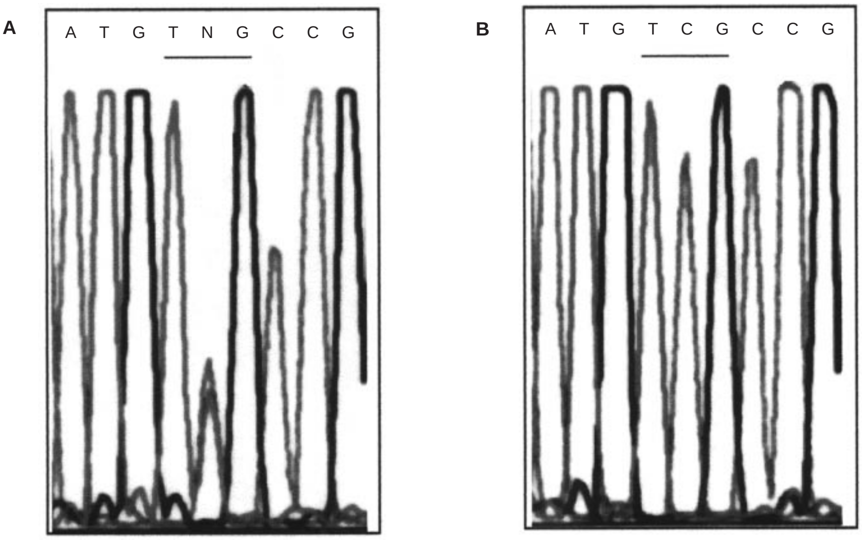

**Figure 1** *p73* sequencing profiles of genomic tumour DNA (case 4) showing the heterozygous mutation at codon 110 (TCG→TTG) (**A**) and of the constitutional DNA (**B**)

### RESULTS

### Mutation analysis of *p73* and *TP53* in MCCs cDNA of five MCC cell lines and ten MCC tumours was amplified by RT-PCR and, consequently, cycle-sequenced using specific nested PCR primers for the detection of mutations in *p73*. In addition, allelic expression for *p73* was determined based upon the known linked polymorphisms in exon 2. The sequencing results and allelic expression data are summarized in Table 1. A C to T mutation at codon 110 was found in one out of ten MCC tumours for *p73*, resulting in a serine to leucine substitution. This nucleotide exchange was also present in the genomic DNA of the tumour but not in the constitutional DNA (Figure 1). No mutations were found in the cell lines. Silent nucleotide substitutions at codon 336 (GCC to GCT) and codon 349 (CAT to CAC) located in exon 9 and at codon 557 (GCG to GCA) and at codon 610 (GCG to GCA) located in exon 14 were found in four (40%) of the ten examined tumours. These polymorphisms were also found in the constitutional patient DNA.

The same panel of MCC cell lines and MCC tumours was also examined for mutations in the *TP53* gene. Four *TP53* mutations were found in two out of five MCC cell lines and one non-sense mutation was observed in one MCC tumour (see Table 1).

### DISCUSSION

Thus far, mutation analyses for *p73* have been performed in several types of carcinomas, oligodendrogliomas and neuroblastomas (Kovalev et al, 1998; Mai et al, 1998*a*, Nimura et al, 1998; Nomoto et al, 1998; Sunahara et al, 1998; Takahashi et al, 1998; Ichimiya et al 1999; Mihara et al, 1999). No mutations were found except for one somatic and one germline mutation in a series of 140 neuroblastomas (Ichimiya et al, 1999). Like MCC, neuroblastoma is a tumour of neuroendocrine origin frequently carrying distal 1p deletions. The two mutations found in neuroblastoma (P405R and P425L) were located within the COOH-terminal region of *p73* (Ichimiya et al, 1999). Recently, Takada et al (1999) showed that the COOH terminus of *p73* had a transactivation function whose activity was significantly reduced by the mis-sense mutations detected in the neuroblastoma tumours.

In the present study, one mis-sense mutation of *p73* in one out of ten MCC tumours was found: a heterozygous C to T transition at nucleotide 439, resulting in a serine to leucine substitution at codon 110 within exon 4. In comparison to the mutations observed in neuroblastoma, this mutation was located in the $NH_2$-terminal region (residues 1–112) of *p73*. The transcriptional transactivation function of the $NH_2$-terminal region of *p73* was shown to have an activity as strong as that of *TP53* (Takada et al, 1999). Further analysis may show that the observed mis-sense mutation in MCC leads to a reduction or complete loss of the transactivation function of the $NH_2$-terminal region of *p73*. The MCC tumour carrying the mutated *p73* allele still expresses the remaining wild-type allele. Similar to *TP53*, this mis-sense mutation might exert a dominant negative effect by an increased stability of the mutant protein compared to that of wild-type protein. Further studies are needed to verify this hypothesis.

The finding of *p73* mutations in neuroblastoma and MCC may also stimulate mutation analyses in other tumour types. Although the low frequency of mutations in both tumours seems to exclude a direct role for *p73* in tumorigenesis, other mechanisms than mutations in *p73* may lead to tissue-specific up- or down-regulation of the gene. In this respect, the available data on allelic expression of *p73* are conflicting. Nomoto et al (1998) found biallelic expression of *p73* in normal lung tissue and distinct patterns of allelic expression in different normal tissues. In contrast, Mai et al (1998*b*) reported monoallelic expression in normal lung tissue and increased and biallelic expression in lung tumour samples. Ichimiya et al (1999) found low *p73* expression in neuroblastoma, while *p73* transcripts were easily detectable in breast carcinoma and colorectal carcinoma under the same conditions. Although we performed no quantification of the *p73* mRNA, we observed biallelic expression in one MCC cell line and eight MCC primary tumours. Clearly, further studies on *p73* expression in normal and cancerous tissues is warranted.

As yet, only one study investigated the possible role of *TP53* in MCC (Schmid et al, 1997). Using immunohistochemical staining, TP53 expression was studied in 25 Merkel cell carcinomas. Only five tumours showed 5–10% TP53-immunoreactive tumour nuclei. Single-strand conformation polymorphism (SSCP) analysis

*© 2000 Cancer Research Campaign* 

for exons 4–8 did not reveal mutations in these five tumours which lead the authors to conclude that *TP53* alterations play only a minor part in the genesis of MCC. However, using this approach it could not be ruled out that the presence of mutations in the other exons of *TP53* were missed. Also, mis-sense and non-sense mutations not leading to accumulation of the gene product would be overlooked in those cases not investigated by SSCP analysis. We also investigated *TP53* for the presence of mutations by sequencing of the entire coding region of the *TP53* gene. *TP53* mutations were found in two out of five MCC cell lines and in one out of ten tumours. In cell line MCC13, three mutations were found at dipyrimidine sites, namely a double base pair C to T transition at nucleotides 857, 858 and a single base pair C to T transition at nucleotide 967 both leading to amino acid substitutions at codon 241 and codon 278 respectively (Table 1). These type of mutations are characteristically induced by absorption of UV irradiation by the DNA (Brash et al, 1991). The mutation found in MCC14/2 is a hemizygous T to A transversion at nucleotide 950 since no wild-type sequence was present. This point mutation is translated into an amino acid substitution from valine to glutamic acid at codon 272. The amino acid changes at codon 241, 272 and 278 reside in mutational hotspots of the sequence-specific DNA-binding domain of the TP53 protein. Alterations in these residues of the TP53 protein will typically result in defective contacts with the DNA and finally lead to the loss of the ability of TP53 to act as a transcription factor. For the particular amino acid substitution at residue 241, Cho et al (1994) could show that DNA contact was not possible as the defective TP53 protein was unable to make contact with the phosphate backbone in the major groove of the DNA. The non-sense mutation at nucleotide 550 in the MCC tumour (case 4) predicted the generation of a truncated TP53 protein. Accumulation of truncated TP53 variants can result in a dominant negative effect and loss of normal TP53 function.

In conclusion, we describe the finding of a sporadic *p73* $NH_2$-terminal located mis-sense mutation in MCC together with the observation of two previously reported loss of function mutations within the COOH-terminal transactivation region of *p73* in neuroblastoma. These mutations provide circumstantial evidence for a role of *p73* in these malignancies of neuroectodermal origin. At present, the exact physiological function of *p73* in tumour development is unclear. In view of these findings, we propose further investigations of *p73* in normal development and tumorigenesis. Further insights in its role in apoptosis (Jost et al, 1997) and possibly other mechanisms such as cell cycle control and cell differentation may provide additional clues for possible contribution of *p73* in tumorigenesis. This report also shows for the first time the occurrence of inactivating *TP53* mutations in 19% of the investigated MCCs. The C to T mutations in cell line MCC13 are known to be caused by UVB irradiation and thus provide further evidence for sun-exposure in some Merkel cell carcinomas.

### ACKNOWLEDGEMENTS

We thank Drs L Kelly and O Williams for providing tumour material (tumour 13 and tumour 14). Dr HJ Cooke and Dr M Litt for providing us with DNA probes pUC1.77 and p1–79 respectively. This work was supported by GOA-grant 12051397, the John A Wiebe Children's Health Care Fund, the Queensland Cancer Fund and the Queensland Radium Institute. Nadine Van Roy is a post-doctoral researcher of the Fund for Scientific Research, Flanders.

### REFERENCES

Brash DE, Rudolph JA, Simon JA, Lin A, McKenna GJ, Baden HP, Halperin AJ and Ponten J (1991) A role for sunlight in skin cancer: UV-induced *p53* mutations in squamous cell carcinoma. *Proc Natl Acad Sci USA* **88:** 10124–10128

Cho Y, Gorina S, Jeffrey PD and Pavletich NP (1994) Crystal structure of a *p53* tumour suppressor–DNA complex: understanding tumorigenic mutations. *Science* **265**: 346–355

Ichimiya S, Nimura Y, Kageyama H, Takeda N, Sunahara M, Shishikura T, Nakamura Y, Sakiyama S, Seki N, Ohira M, Kaneko Y, McKeon F, Caput D and Nakagawara A (1999) *p73* at chromosome 1p36 is lost in advanced stage neuroblastoma but its mutation is infrequent. *Oncogene* **18**: 1061–1066

Jost CA, Marin MC and Kaelin WG Jr (1997) p73 is a human p53-related protein that can induce apoptosis. *Nature* **389**: 191–194

Kaghad M, Bonnet H, Yang A, Creancier L, Biscan JC, Valent A, Minty A, Chalon P, Lelias JM, Dumont X, Ferrara P, McKeon F and Caput D (1997) Monoallelically expressed gene related to *p53* at 1p36, a region frequently deleted in neuroblastoma and other human cancers. *Cell* **90**: 809–819

Kovalev S, Marchenko N, Swendeman S, LaQuaglia M and Moll UM (1998) Expression level, allelic origin, and mutation analysis of the *p73* gene in neuroblastoma tumours and cell lines. *Cell Growth Diff* **9**: 897–903

Mai M, Huang H, Reed C, Qian C, Smith JS, Alderete B, Jenkins R, Smith DI and Liu W (1998*a*) Genomic organization and mutation analysis of *p73* in oligodendrogliomas with chromosome 1 p-arm deletions. *Genomics* **51**: 359–363

Mai M, Yokomizo A, Qian C, Yang P, Tindall DJ, Smith DI and Liu W (1998*b*) Activation of *p73* silent allele in lung cancer. *Cancer Res* **58**: 2347–2349

Mihara M, Nimura Y, Ichimiya S, Sakiyama S, Kajikawa S, Adachi W, Amano J and Nakagawara A (1999) Absence of mutation of the *p73* gene localized at chromosome 1p36.3 in hepatocellular carcinoma. *Br J Cancer* **79**: 164–167

Mitelman F, Johansson B and Mertens F (1998) *Catalog of Chromosome Aberrations in Cancer '98*. Version 1. Wiley-Liss: New York (CD-rom)

Nimura Y, Mihara M, Ichimiya S, Sakiyama S, Seki N, Ohira M, Nomura N, Fujimori M, Adachi W, Amano J, He M, Ping YM and Nakagawara A (1998) *p73*, a gene related to *p53*, is not mutated in esophageal carcinomas. *Int J Cancer* **78**: 437–440

Nomoto S, Haruki N, Kondo M, Konishi H, Takahashi T, Takahashi T and Takahashi T (1998) Search for mutations and examination of allelic expression imbalance of the *p73* gene at 1p36.33 in human lung cancers. *Cancer Res* **58**: 1380–1383

Ratner D, Nelson BR, Brown MD and Johnson TM (1993) Merkel cell carcinoma. *J Am Acad Dermatol* **29**: 143–156

Schmid M, Janßen K, Dockhorn-Dworniczak B, Metze D, Zelger BW, Luger TA and Schmid KW (1997) *p53* abnormalities are rare events in neuroendocrine (Merkel cell) carcinoma of the skin. An immunohistochemical and SSCP analysis. *Virchows Arch* **430**: 233–237

Schwab M, Praml C and Amler LC (1996) Genomic instability in 1p and human malignancies. *Genes Chromosomes Cancer* **16**: 211–229

Sunahara M, Ichimiya S, Nimura Y, Takada N, Sakiyama S, Sato Y, Todo S, Adachi W, Amano J and Nakagawara A (1998) Mutational analysis of the *p73* gene localized at chromosome 1p36.3 in colorectal carcinomas. *Int J Oncol* **13**: 319–323

Takada N, Ozaki T, Ichimiya S, Todo S and Nakagawara A (1999) Identification of a transactivation activity in the COOH-terminal region of *p73* which is impaired in the naturally occurring mutants found in human neuroblastomas. *Cancer Res* **59**: 2810–2814

Takahashi H, Ichimiya S, Nimura Y, Watanabe M, Furusato M, Wakui S, Yatani R, Aizawa S and Nakagawara A (1998) Mutation, allelotyping, and transcription analyses of the *p73* gene in prostatic carcinoma. *Cancer Res* **58**: 2076–2077

Van Gele M, Van Roy N, Ronan SG, Messiaen L, Vandesompele J, Geerts ML, Naeyaert JM, Blennow E, Bar-Am I, Das Gupta TK, van der Drift P, Versteeg R, Leonard JH and Speleman F (1998) Molecular analysis of 1p36 breakpoints in two Merkel cell carcinomas. *Genes Chromosomes Cancer* **23**: 67–71

Van Roy N, Laureys G, Cheng NC, Willem P, Opdenakker G, Versteeg R and Speleman F (1994) 1;17 translocations and other chromosome 17 rearrangements in human primary neuroblastoma tumours and cell lines. *Genes Chromosomes Cancer* **10**: 103–114

 *© 2000 Cancer Research Campaign*
